# Dissecting multimodal predictors of psychosis risk and remission in youth at ultra‑high risk of developing psychosis: An exploratory study

**DOI:** 10.1371/journal.pmen.0000686

**Published:** 2026-08-21

**Authors:** Weijia Kong, Samuel Ming Xuan Tan, Matheus Calvin Lokadjaja, Jordon Junyang Kho, Zixu Yang, Jieyin Yee, Max Lam, Jimmy Lee, Wilson Wen Bin Goh

**Affiliations:** 1 Lee Kong Chian School of Medicine, Nanyang Technological University, Nanyang Ave, Singapore; 2 School of Biological Sciences, Nanyang Technological University, Nanyang Ave, Singapore; 3 Institute of Mental Health, Singapore, Singapore; 4 Centre for Biomedical Informatics, Nanyang Technological University, Nanyang Ave, Singapore; 5 Centre of AI in Medicine, Nanyang Technological University, Nanyang Ave, Singapore; 6 Division of Neurology, Department of Brain Sciences, Faculty of Medicine, Imperial College London, London, United Kingdom; Instituto Federal do Maranhão: Instituto Federal de Educacao Ciencia e Tecnologia do Maranhão, BRAZIL

## Abstract

Early identification of individuals at risk of developing psychosis enables timely intervention and better clinical outcomes. Current approach relies on clinical assessments, such as the Comprehensive Assessment for At‑Risk Mental States (CAARMS), which provides an Ultra‑High‑Risk (UHR) classification but have limited predictive precision. Artificial intelligence (AI) models integrating neuroimaging, clinical, genetic, and biomolecular data show promise for improving prognostic accuracy, yet their clinical applicability remains uncertain. We systematically evaluated the contribution of six data modalities, ranging from socio‑environmental factors to polygenic risk scores, using data from 56 UHR participants in the Longitudinal Youth at‑Risk Study (LYRIKS) cohort. Modalities were iteratively included and excluded to generate candidate models tested on two tasks: (1) predicting transition to psychosis within 12 months and (2) predicting remission from UHR status within the same period. Statistical significance was assessed via permutation testing. Of the 56 UHR participants, 12 developed psychosis and 26 achieved remission from UHR status. The model combining CAARMS, socio-environmental risk factors, and social functioning (CAARMS + RISK + HiSoC) achieved the best overall performance for both predicting conversion (MCC = 0.71, SP = 0.93, SE = 0.78) and remission (MCC = 0.64, SP = 0.82, SE = 0.77), and was the only model to significantly outperform null models in the conversion task. Behavioural and socio‑environmental modalities demonstrate strong predictive value for forecasting outcomes in UHR within multimodal AI frameworks. While molecular and genetic modalities remain promising, further advances are needed to translate their high‑dimensional complexity into clinically useful predictive tools.

## Introduction

The early identification of individuals at elevated risk for psychosis represents a critical step toward improving clinical outcomes and guiding timely intervention. [1]. Current approaches largely rely on psychometric instruments such as the Comprehensive Assessment for At-Risk Mental States (CAARMS) [2] and Structure Interview for Prodromal Syndrome (SIPS) [3] to identify Ultra-High-Risk (UHR) individuals. However, these psychometric instruments tend to have low specificity [4].

Various measurements with appreciable correlation to clinical outcomes have been proposed to supplement psychometric assessment. This includes polygenic risk scores (PRS) [5], cognition [6,7], social functioning [8,9], familial, environmental, and life history factors including advanced parental age [10,11], family history of psychiatry disorder [12], education attainment [13,14], adolescent smoking [15], peripheral blood biomarkers [16], and electronic health records [17]. Efforts to combine these modalities using AI and machine learning (ML) methods are promising, often reporting excellent performances [16–19]. Therefore, it is foreseeable that multimodal prediction models will persist as a prominent trend in the future [20]. However, deployment practicability is another matter, and requires careful evaluations on generalizability, resource cost, and data availability [21,22].

Multimodal models typically employ variables derived from 3 to 8 assessments (or modalities) [19,23–25]. Employing a large number of data modalities can substantially limit the practicality of predictive models in clinical settings. To acquire the required measurements, clinicians may either conduct all assessments in full, which is logistically demanding, or selectively perform only those components necessary for generating model inputs. While the latter approach reduces operational burden, isolated variables obtained without contextual information hold limited interpretive value for clinical decision-making. Moreover, the diversity of modalities complicates validation and comparative efforts, as studies seeking to evaluate multiple models concurrently must perform numerous assessments on large cohorts to capture the full spectrum of measurements used across models. Such undertakings are resource‑intensive and financially prohibitive, posing significant barriers to clinical translation.

The relative contribution of individual modalities to psychosis prediction remains poorly characterized, and no consensus exists on an optimal combination. Rather than testing a single pre-specified model, we take an exploratory, modality-centric approach: features from each modality are treated as predefined modules and combined systematically to generate candidate models, enabling a direct comparison of how each data source contributes to predictive performance. We propose an exploratory approach to evaluate modality importance in predictive models. Our modality-centric approach treats features from each modality as “predefined modules”, which are combined systematically to generate a set of candidate models, which are then evaluated for predictive performance. We test this approach using data from the Longitudinal Youth at-Risk Study (LYRIKS) cohort [26]. Models are evaluated on 2 key tasks: 1) identifying pre-conversion individuals from individuals who do not convert to psychosis (PRE-CVT vs NON-CVT) and 2) identifying individuals who remit from UHR status from those who do not (PRE-RMT vs NON-RMT).

## Methods

### Ethics statement

Ethics approval was obtained from the National Healthcare Group Domain Specific Review Board (2009/00167), and written informed consent was obtained from all participants.

### Participants

Participants were recruited between 25 June 2009 and 11 November 2012 from LYRIKS, an observational cohort involving individuals aged 14–29 years, designed to identify and characterize risk factors associated with the development of psychosis. (Lee et al., 2013). As genotyping (polygenic risk score, PRS) and RNA-Seq data were available only for a subset of LYRIKS participants, study sample had to be unfortunately restricted to individuals with complete data for both modalities, resulting in a final cohort of 56 participants (41 male, 15 female) with a mean age of 21.7 years (SD = 3.06).

### Data collection

Six modalities collected from the same cohort as part of the LYRIKS study [26] were evaluated:

- Comprehensive Assessment for At-Risk Mental State (CAARMS) [2], an assortment of risk variables of psychotic disorders (RISK), [27]
- Neurocognition on the Brief Assessment of Cognition in Schizophrenia (BACS) [28]
- Social functioning on the High-Risk Social Challenge (HiSoC) [27]
- Genetics via the PRS for schizophrenia
- RNA-sequencing (RNA-SEQ) of Peripheral blood mononuclear cells (PBMCs)

Measurements in the LYRIKS study were collected at six-month intervals over a two-year period. PRE-CVT samples refer to measurements obtained within 12 months preceding conversion to psychosis. NON-CVT samples correspond to baseline assessments from participants who did not convert to psychosis. PRE-RMT samples represent baseline measurements from individuals who subsequently remitted from UHR status, while NON-RMT samples comprise both PRE-CVT and NON-CVT measurements from participants who did not achieve remission from the UHR state

CAARMS variables include the different subgroups of UHR namely Genetic Vulnerability (VUL), Brief Limited Intermittent Psychotic Symptoms (BLIPS), Attenuated Psychosis Symptoms (APS) along with the subjective distress measures for Unusual Thought Content Distress (UTC Distress), Perceptual Abnormality Distress (PA Distress), Non-Bizarre Ideas Distress (NBI Distress), and Disorganized Speech Distress (DS Distress) [2].

RISK variables are socio-environmental features associated with schizophrenia and psychosis. This includes parental age at birth [10,11], family history of psychiatric disorder [12], education attainment [13,14], and adolescent smoking [15]. We also included *history of depressive disorders, defined* as a binary indicator (0/1) lifetime prevalence of major depressive disorder, dysthymia, or depressive disorder not otherwise specified as assessed on the Structured Clinical Interview for DSM-IV-TR (SCID-IV-TR). The SCID-IV-TR is a widely used semi-structured diagnostic tool to ascertain psychiatric disorders. Parental age at birth was collected in the form of *maternal age at birth* and *paternal age at birth*. Education attainment was collected in the form of *Late Primary School Leaving Examination (PSLE)* and *low education relative to age*. The PSLE is a mandatory national examination taken by all school children at the age of 12 in Singapore. We defined an individual to have Late PSLE if they undertook PSLE after the age of 13. Individuals were indicated as having Low education relative to age if they have not attained or are currently undergoing post-secondary education by age 18. Adolescent smoking was collected in the form of *Childhood and Adolescent Smoking*, defined as tobacco use before the age of 18.

BACS variables included the scores for Verbal Memory, Verbal Fluency, Token Motor Task, Digit Sequencing, Symbol Coding and Tower of London [28].

HiSoC variables consist of composite scores based on factors identified in [27] namely: Social-interpersonal (Social), affect (Affect), odd behaviour and speech (Odd behaviour) were used.

The PRS score is the weighted sum of risk alleles using GWAS summary statistics from the PGC East-Asian schizophrenia meta-analysis (PGC SCZ-EAS) [29] as discovery samples.

RNA-SEQ data capturing the gene expression profiles of PBMCs were aligned to the Ensembl GRCh38 using STAR 2.7.9a [30]. Aligned reads are quantified using featureCount from the Subread 2.0.3 R Package [31]. To deal with high-dimensionality issue, we reviewed current literature to obtain a list of candidate genes and selected the top 5 genes exhibiting the greatest effect size between samples collected before psychosis conversion and samples collected after conversion (Tables A and B in S1 Appendix). The selection of 5 genes is not merely arbitrary --- this is to match the average number of variables in other modalities. The 5 genes selected are CCDC134, BRCA1, EGR1, NLN, and TOX. Processing details for PRS and RNA-seq data are available in S1 Appendix.

### Data pre-processing

Missing data in cognitive measurements were imputed using Multivariate Imputation by Chain Equation (MICE). via the miceRanger R package [32]. with mean matching as the value selector and 15 iterations. Controls and UHR participants were imputed separately to prevent information leakage between groups. A single imputation (m = 1) was performed. The majority of variables had no missing data; missingness was concentrated in HiSoC social functioning scores (35–37% in UHR, 30% in controls), with minimal missingness in other variables (<5%). To assess the robustness of results to imputation uncertainty, a sensitivity analysis was conducted by repeating the imputation 10 times independently and re-evaluating model performance across the resulting datasets (S1 Fig). MICE was chosen due to its previously demonstrated good performance in comparable studies [18,33].

RNA-SEQ data was obtained from part of our previous study [34]. Raw RNA count data was normalized for library size and corrected for mean-variance dependence using the rlog method from the DESEQ2 package [35]. The correction was done in a non-blinded manner to account for the possibility that different classes have different mean-variance dependencies.

A full list of variables and respective abbreviations is listed in (Table C in S1 Appendix).

### Data modelling

Support Vector Machines (SVMs) are proven and reliable models for the recognition of subtle patterns in complex datasets [36]. For our modelling, we employed an SVM with a linear kernel and an L2 penalty as a measure to minimize overfitting. Class weights were adjusted to be inversely proportional to class frequencies to control for class imbalances. To optimize model performance, we conducted a grid search for hyperparameter tuning, exploring a range of values for the regularization parameter C recommended by optimization studies [37], including {2^-5^, 2^-3^, 2^-1^, 2, 2^3^, 2^5^, 2^-7^, 2^-9^, 2^-11^}. The evaluation of the model’s performance was carried out using Matthew’s Correlation Coefficient (MCC), Sensitivity (SP), and Specificity (SE). MCC was selected as the primary metric because it incorporates all four quadrants of the confusion matrix (TP, TN, FP, FN) into a single balanced measure, making it particularly suitable for imbalanced classification settings such as the PRE-CVT task (12 vs 44) [38,39]. MCC evaluates performance at the actual decision boundary without requiring probabilistic outputs, and penalises classifiers that favour the majority class, avoiding the inflated scores that can arise with accuracy or AUROC under imbalanced conditions.


$$\begin{array}{c} MCC=\frac{TP*TN-FP*FN}{\sqrt{\left(TP+FP)(TP+FN)(TN+FP)(TN+FN\right)}} \end{array}$$

$$\begin{array}{c} True\;Positive\;Rate=Sensitivity=Recall=\frac{TP}{TP+FN} \end{array}$$

$$\begin{array}{c} True\;Negative\;Rate=Specificity=\frac{TN}{FP+TN} \end{array}$$

Where TP, FP, TN, and FN correspond to True Positives, False Positives, True Negatives and False Negatives respectively. MCC provides an overall gauge of model performance while SE and SP measure the ability of the model to detect true positives and true negatives respectively.

Bias-corrected values for MCC, SE, and SP were obtained via internal bootstrap validation with refitting [40]. This approach was chosen because the small sample size (n = 56, with only 12 converters) precluded the use of cross-validation, which would yield unstable estimates due to insufficient samples per fold. The modelling procedure is described as follows:

1. At each parameter C, build an SVM using the original dataset. Calculate an initial performance via MCC, sensitivity, and specificity.
2. Take a bootstrap sample, drawing samples with replacements from the original dataset, equal to the number of original samples.
3. Refit the same model on this bootstrap sample and evaluate its performance on the original dataset to obtain an optimism estimate.4. Repeat steps 2–3 for 1000 iterations, generating 1000 bootstrap model performance metrics. The bias is estimated as the mean difference between the apparent performance and the bootstrap performance. The bias-corrected metric is then obtained by subtracting the absolute bias from the apparent performance.A total of 20 models were evaluated. Initially, five base models were tested, each constructed from the variables of a single modality: CAARMS, RISK, HiSoC, BACS, and RNA-SEQ. The PRS modality was excluded from base model testing due to numerical instability encountered during permutation testing on one-dimensional data. Subsequently, five models were evaluated, each combining CAARMS variables with one additional modality, and ten models were tested that combined CAARMS with two additional modalities (see Table D in S1 Appendix). To minimize overfitting risk given the modest sample size (curse-of-dimensionality), models incorporating more than two additional modalities were not examined.

### Permutation test

The statistical significance of each model’s ability to discriminate between UHR individuals with differing outcomes was assessed using permutation testing with 5,000 iterations. For the five base models, null models were generated by replacing modality variables with an equal number of random variables sampled from parametric distributions, while retaining the original sample size (n = 56) and outcome labels: continuous variables were drawn from the standard normal distribution N(0,1) and binary variables from the binomial distribution B(1, p), where p matched the proportion of positive values in the RISK and CAARMS modalities (0.265 and 0.304, respectively).

Only RISK and CAARMS included binary variables. For the remaining 15 combination models, null models were constructed by training on the CAARMS variables plus a matched number of random variables for each non-CAARMS component, sampled as above. For instance, the null for the CAARMS  +  BACS model comprised 8 CAARMS variables plus 6 random continuous variables.

The empirical p-value is defined as the probability of a null model obtaining performance (MCC) greater or equal to the performance achieved by the actual model and defined as:


$$\begin{array}{c} p_{MCC}=\frac{number\;of\;times\;MCC_{null}\geq \;MCC_{actual}}{\text{5}\text{0}\text{0}\text{0}} \end{array}$$

The empirical p-values for SE and SP are calculated in the same manner. A model is defined to strongly outperform, moderately outperform, and weakly outperform null models if p < 0.01, p < 0.05, and p < 0.10 respectively.

## Results

### Participant demographics

Of the 56 UHR participants included in the cohort, 12 individuals converted to psychosis with a mean time to conversion of 12.8 months. Among the remaining 44 participants, 26 achieved remission from UHR status, while 18 maintained UHR status throughout the follow-up period. No statistically significant differences in age or sex distributions were observed between groups (see Table 1).

**Table 1:** Participant demographic and variables used.

	Mean [SD] or N [%]				
Variable	Pre-convert (n = 12)	Pre-remit (n = 26)	Maintain (n = 18)	ANOVA or Chi2 p-val	Adjusted p-val
					
**Female**	2 [16.7]	7 [26.9]	6 [33.3]	0.6	0.74
					
**Age, years**	21.3 [3.31]	22.5 [2.9]	20.9 [3.38]	0.229	0.489
**CAARMS**					
VUL	3 [25.0]	7 [26.9]	11 [61.1]	0.0424	0.182
APS	10 [83.3]	21 [80.8]	12 [67.0]	0.46	0.609
BLIPS	0 [0]	4 [15.4]	0 [0]	0.0833	0.283
UTC distress	21.3 [26.6]	30.0 [36.8]	13.9 [28.31]	0.287	0.507
NBI distress	72.1 [25.9]	54.4 [36.0]	30.3 [31.73]	0.0048	0.073
PA distress	58.8 [36.1]	41.5 [36.3]	15.6 [27.13]	0.0042	0.073
DS distress	8.33 [20.3]	9.42 [26.5]	3.89 [13.8]	0.715	0.818
**BACS**					
Verbal Memory	41.3 [8.07]	45.8 [11.2]	48.1 [8.91]	0.212	0.489
Digit Sequencing	18.0 [3.16]	19.9 [4.9]	19.9 [3.51]	0.396	0.609
Token Motor Task	64.7 [16.3]	72.9 [10.1]	78.4 [10.3]	0.014	0.089
Verbal Fluency	35.4 [8.62]	48.1 [14.7]	48.4 [10.6]	0.012	0.089
Symbol Coding	49.9 [14.1]	58.9 [10.4]	61.4 [9.74]	0.026	0.119
Tower of London	16 [2.92]	18.4 [2.40]	17.88 [2.23]	0.027	0.119
**HiSoC**					
Affect	17.6 [3.63]	20.9 [2.79]	20.9 [2.94]	0.0070	0.089
Odd behaviour	16.5 [3.03]	18.3 [2.23]	18.0 [3.22]	0.192	0.489
Social	12.5 [2.59]	14.1 [1.87]	13.6 [2.89]	0.204	0.357
**Risk Variables**					
History of depressive disorder	5 [41.7]	12 [46.2]	5 [27.8]	0.463	0.609
Family history of depressive and anxiety disorder	2 [16.7]	3 [11.5]	7 [38.9]	0.085	0.283
Family history of schizophrenia or psychosis	5 [41.7]	12 [46.2]	8 [44.4]	0.967	0.967
Maternal age at birth	31.7 [4.5]	29.3 [6.0]	31.1 [7.01]	0.466	0.609
Paternal age at birth	31.2 [5.02]	31.0 [7.2]	35.1 [8.42]	0.271	0.507
Low education attainment relative to age	4 [33.3]	4 [15.4]	3 [16.7]	0.402	0.609
Late graduation from primary education	2 [16.7]	0 [0]	2 [11.1]	0.131	0.357
Childhood or adolescent smoker	5 [41.7]	8 [30.8]	2 [11.1]	0.148	0.37
**RNA markers^1^**					
CCDC134	10.2 [0.238]	10.3 [0.231]	10.1 [0.168]	0.451	0.609
BRCA1	9.41 [0.220]	9.40 [0.223]	9.42 [0.228]	0.956	0.967
EGR1	8.22 [0.317]	8.26 [0.389]	8.22 [0.319]	0.937	0.967
NLN	9.79 [0.187]	9.82 [0.211]	9.77 [0.167]	0.736	0.818
TOX	10.0 [0.193]	10.1 [0.228]	10.1 [0.246]	0.617	0.740

^1^Values are DESEQ2 rlog quantities

Nominally significant between-group differences (p < 0.05) were identified in NBI distress, PA distress, BACS token motor task, BACS verbal fluency, BACS symbol coding, and BACS Tower of London assessments. However, after correction for multiple testing, none of these variables retained statistical significance across groups.

### Performance of base models

**Fig 1:**
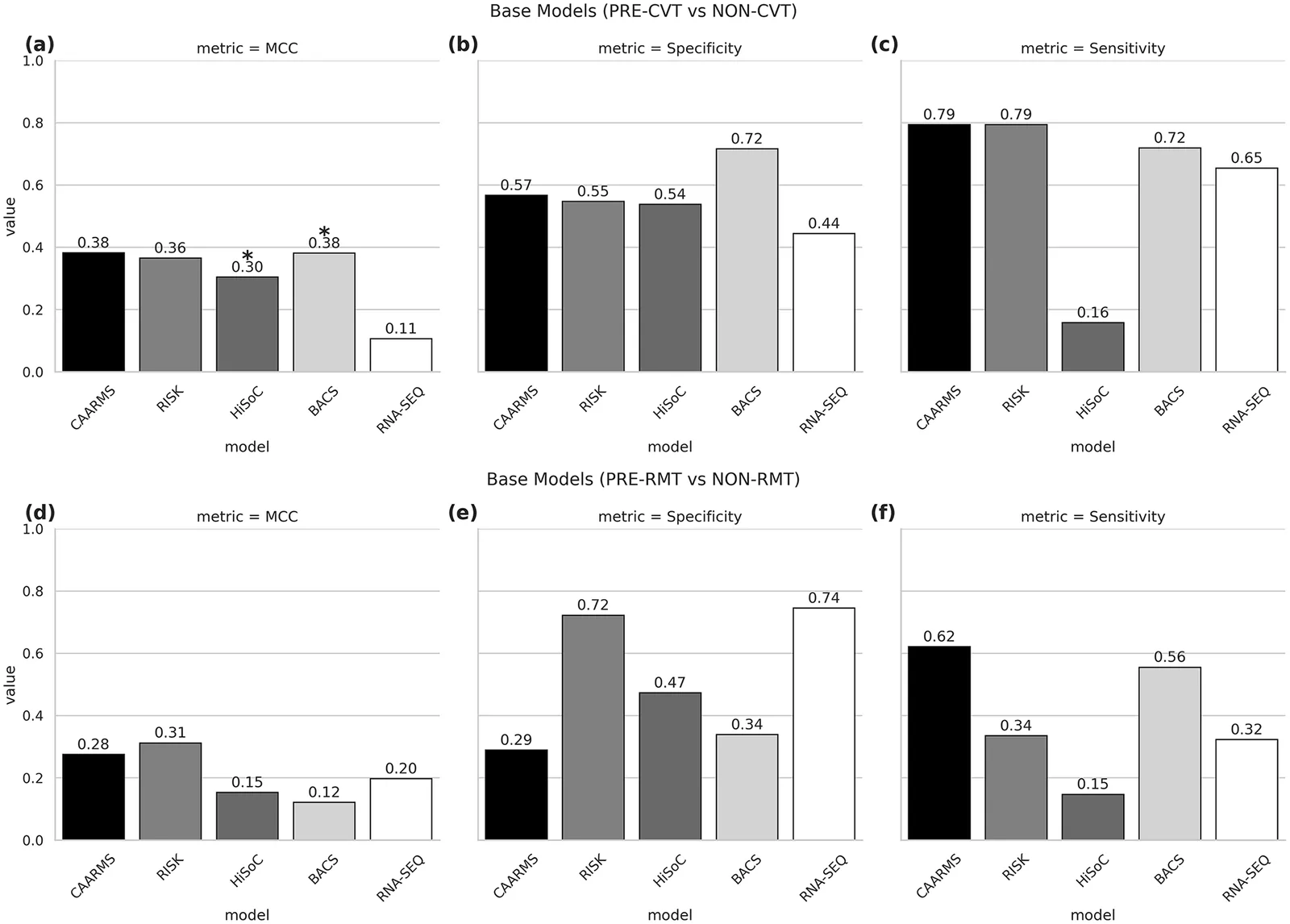
Performance of base models Bar plots of the (a) MCC, (b) SP and (c) SE for each CAARMS + 2 modality model in the PRE-CVT vs NON-CVT tasks as well as the bar plots of the (d) MCC, (e) SP and (f) SE for the models in the PRE-RMT vs non- remit tasks. An asterisk (*) indicates that the model outperformed null models. The MCC of the HiSoC and BACS models outperform null models at p = 0.057 and p = 0.06 respectively.

We first examined prediction performance of models trained using variables from each modality in isolation in the PRE-CVT vs NON-CVT task. The highest MCC was tied between the CAARMS (MCC = 0.38, SP = 0.57, SE = 0.79) and BACS (MCC = 0.38, SP = 0.72, SE = 0.72) models. The highest SP = 0.72 was achieved by the BACS model. The highest SE was tied between the CAARMS model and the RISK model (MCC = 0.37, SP = 0.55, SE = 0.79). The RNA-SEQ model performed the worst in terms of MCC and SP (MCC = 0.11, SP = 0.44, SE = 0.65) while the HiSoC model (MCC = 0.30, SP = 0.54, SE = 0.16) performed the worst in terms of SE. The MCC of the HiSoC and BACS models weakly outperformed null models at p = 0.057 and p = 0.060 respectively. No model outperformed null models in SP and SE (Fig 1A–C).

In the PRE-RMT vs NON-RMT task, the MCC was achieved by the RISK model (MCC = 0.31, SP = 0.72, SE = 0.34). The highest SP was achieved by the RNA-SEQ model (MCC = 0.20, SP = 0.74, SE = 0.32), and the highest SE was achieved by the CAARMS model (MCC = 0.28, SP = 0.29, SE = 0.62). The BACS model performed the worst in terms of MCC, SP, and SE (MCC = 0.12, SP = 0.34, SE = 0.56). No model outperformed null models in MCC, SP, and SE (Fig 1D–F).

A summary of performance metrics and corresponding empirical p-values for all base models across both tasks is presented in Table E in S1 Appendix. Model coefficients for each evaluation are detailed in Table F in S1 Appendix.

### Performance of CAARMS + 1 modality models

We next examined the performance of models trained using variables from the combinations of CAARMS and 1 other modality.

**Fig 2:**
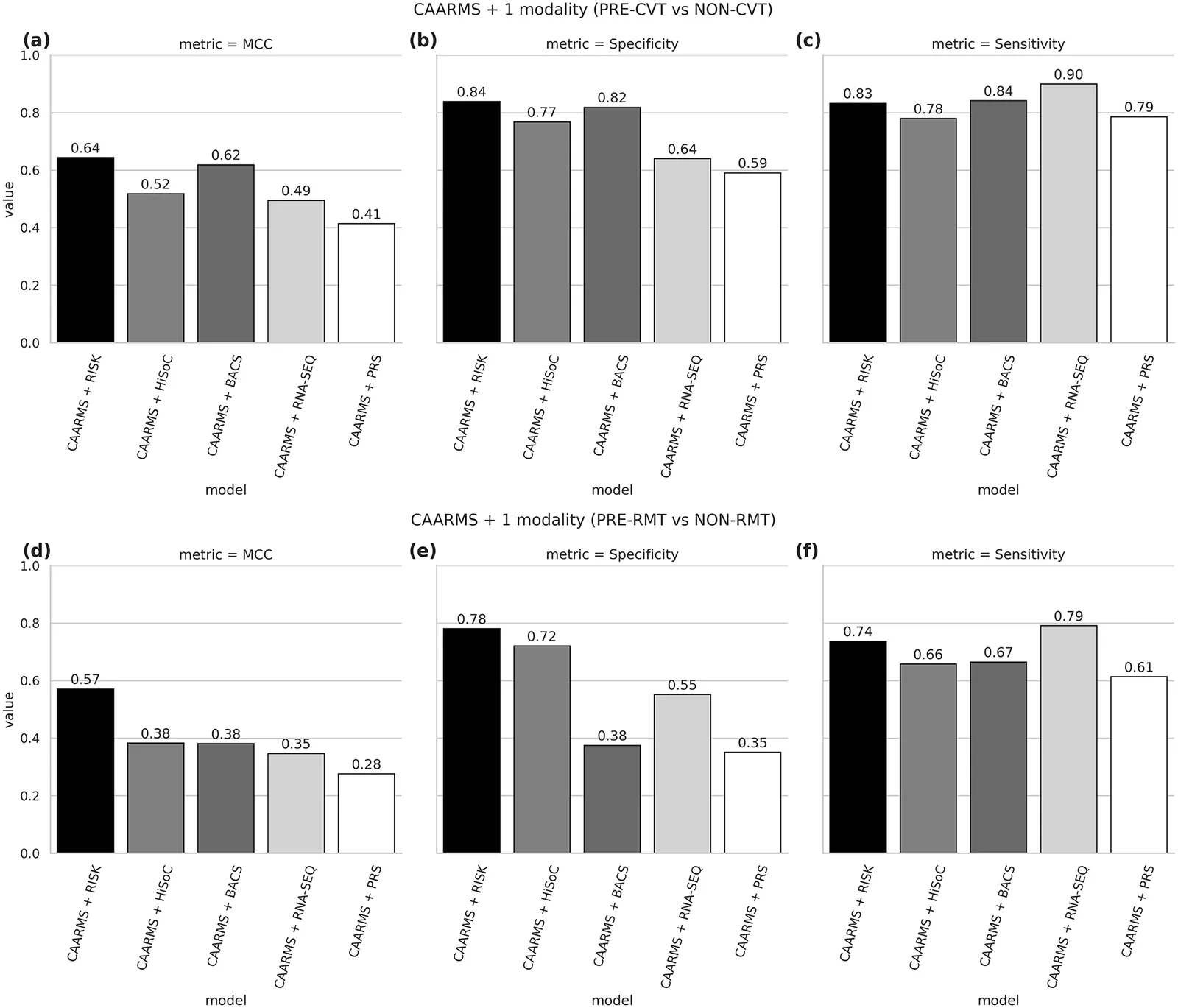
Performance of CAARMS + 1 modality models Bar plots of the (a) MCC, (b) SP and (c) SE for each CAARMS + 1 modality model in the PRE-CVT vs NON-CVT tasks as well as the bar plots of the (d) MCC, (e) SP and (f) SE for the models in the PRE-RMT vs non- remit tasks. An asterisk (*) indicates that the model outperformed null models. None of the models in either task outperformed null models.

In the PRE-CVT vs NON-CVT task, the highest MCC and SP were achieved by the model trained on CAARMS + RISK variables (MCC = 0.64, SP = 0.84, SE = 0.83). the highest SE was achieved by model trained on the CAARMS + RNA-SEQ variables (MCC = 0.50, SP = 0.64, SE = 0.90). The model trained on CAARMS + PRS variables performed the worst in terms of MCC and SP (MCC = 0.41, SP = 0.59, SE = 0.79), while the model trained on CAARMS + HiSoC variables performed the worst in terms of SE (MCC = 0.52, SP = 0.77, SE = 0.78). None of the models outperformed the null models in MCC, SP, and SE (Fig 2A–C).

In the pre-remission vs non-remission task, the highest MCC and SP were also achieved by the model trained on CAARMS + RISK variables (MCC = 0.57, SP = 0.78, SE = 0.74). The highest SE was also achieved by the model trained on CAARMS + RNA-SEQ variables (MCC = 0.35, SP = 0.55, SE = 0.79). The model trained on CAARMS + PRS variables performed the worst in terms of MCC, SP, and SE (MCC = 0.41, SP = 0.59, SE = 0.79). No model outperformed null models in MCC, SP, and SE (Fig 2D–F).

A table of the performance metric and associated empirical p-values for all CAARMS + 1 modality models for both tasks can be found in Table G in S1 Appendix. The coefficients of each model can be found in Table H in S1 Appendix.

### Performance of CAARMS + 2 modalities models

We next examined the performance of models trained using variables from the combinations of CAARMS and 2 other modalities.

**Fig 3:**
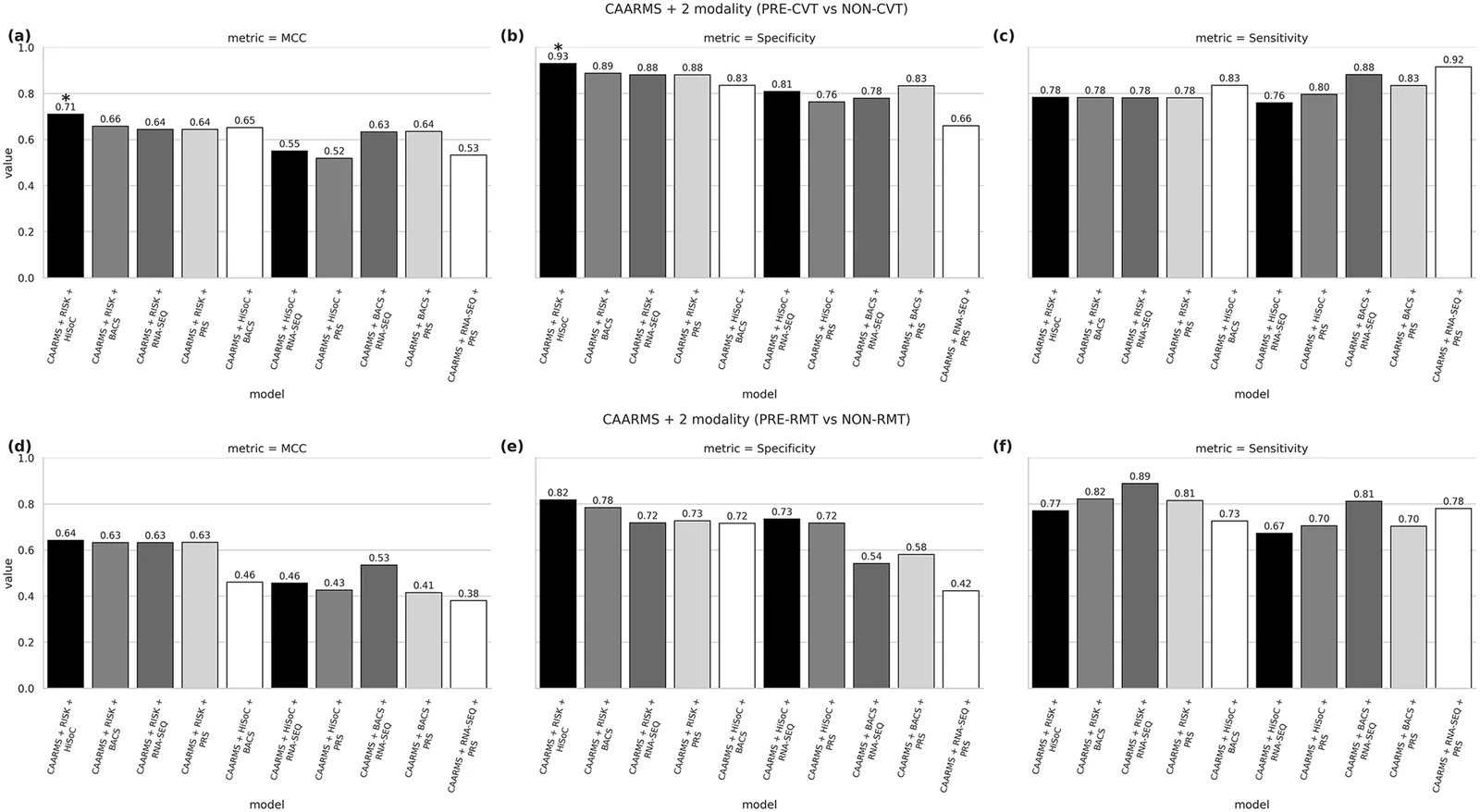
Performance of CAARMS + 2 modality models Bar plots of the (a) MCC, (b) SP and (c) SE for each CAARMS + 2 modality model in the PRE-CVT vs NON-CVT tasks as well as the bar plots of the (d) MCC, (e) SP and (f) SE for the models in the PRE-RMT vs non-remit tasks. An asterisk (*) indicates that the model outperformed null models. Only the MCC and SP for the CAARMS + RISK + HiSoC model outperform null models at p = 0.06 and p = 0.028 respectively.

In the PRE-CVT vs NON-CVT task, the highest MCC and SP were achieved by the model trained on CAARMS + RISK + HiSoC variables (MCC = 0.71, SP = 0.93, SE = 0.78). The highest SE was achieved by the model trained on the CAARMS + RNA-SEQ + PRS variables (MCC = 0.53, SP = 0.66, SE = 0.91). The model trained on CAARMS + HiSoC + PRS variables (MCC = 0.52, SP = 0.76, SE = 0.80) performed the worst in terms of MCC, while the models trained on CAARMS + RNA-SEQ + PRS variables (MCC = 0.53, SP = 0.66, SE = 0.91) and CAARMS + HiSoC + RNA-SEQ variables (MCC = 0.55, SP = 0.81, SE = 0.76) performed worst form SP and SE respectively. The MCC for the CAARMS + RISK + HiSoC model weakly outperformed the null models at p = 0.056 and the SP of the CAARMS + RISK + HiSoC model moderately outperformed the null models at p = 0.028 (Fig 3A–C).

In the pre-remission vs non-remission task, the model trained on CAARMS + RISK + HiSoC variables (MCC = 0.64, SP = 0.82, SE = 0.77) performed the best in terms of MCC and SP while the model trained on CAARMS + RISK + RNA-SEQ variables (MCC = 0.63, SP = 0.72, SE = 0.89) performed the best in terms of SE. The model trained on CAARMS + RNA-SEQ + PRS variables (MCC = 0.38, SP = 0.42, SE = 0.78) performed the worst in terms of MCC and SP while the model trained on CAARMS + HiSoC + RNA-SEQ variables (MCC = 0.46, SP = 0.73, SE = 0.67) performed in the worst in terms of SE. No model outperformed null models (Fig 3D–F).

A table of the performance metric and associated empirical p-values for all CAARMS + 2 modality models in both tasks can be found in Table I in S1 Appendix. The coefficients of each model can be found in Tables J and K in S1 Appendix.

## Discussion

In this exploratory study, we systematically evaluated combinations of various data modalities and their relationships with predictive outcomes to identify which modalities are information-rich and practical for integration into models intended for clinical deployment. Our findings suggest that biology-based measures may require further feature engineering and refined data processing before their full potential is realized. Due to limitations in our dataset size, we advocate for the accumulation of validation results across multiple cohorts to enable meta-analyses that can more comprehensively assess the performance of different modality combinations. Such an approach would support the development of reliable and generalizable predictive models, balanced with logistical considerations such as cost and data accessibility.

Specifically, while many models demonstrated good classification performance, only those based on variables from the BACS, HiSoC, and the combined CAARMS + RISK + HiSoC variable sets significantly outperformed null models—and only for the PRE-CVT versus NON-CVT prediction task. The CAARMS + RISK + HiSoC model achieved the highest Matthews correlation coefficient and specificity, indicating promising prognostic potential when supplementing CAARMS with these modalities.

Models incorporating the CAARMS + RISK + HiSoC variables are particularly promising since RISK variables are routinely collected during clinical assessments, with minimal additional logistical burden beyond administering the single HiSoC assessment. However, education attainment measures used, such as late PSLE and post-secondary education, reflect the Singapore education system and will require standardization for broader applicability. Moreover, Singapore’s uniquely low rates of drug use and adolescent smoking may limit generalizability [41], but comparison with cohorts from other countries could help elucidate how these socio-environmental factors influence mental health disorder progression.

Our finding that socio-environmental risk factors, particularly family psychiatric history and developmental indicators, substantially enhanced prediction when combined with clinical symptoms resonates with calls to move beyond symptom-based DSM and ICD criteria for early identification of psychopathology in high-risk youth. Previous literature argued that accurate psychiatric diagnosis requires looking beyond isolated symptoms to the broader clinical and familial context in which they occur [42]. This perspective is supported by longitudinal family studies spanning six decades, which demonstrated that family psychiatric history, developmental antecedents in offspring, and clinical course patterns are essential for individualized risk stratification in mood and psychotic disorders [43]. In our data, the RISK modality which capture family history of psychiatric disorder, personal history of depression, parental age at birth, and education attainment, contributed the largest incremental gain when added to CAARMS, and the three-modality model incorporating HiSoC achieved the strongest performance overall. These results provide empirical support, from a psychosis-risk cohort, for the principle that comprehensive multi-domain assessment integrating familial, developmental, and social dimensions outperforms approaches relying on clinical symptom measures alone.

We identified several noteworthy observations that challenge the interpretation of seemingly strong classification performance in our prediction models. For example, the BACS and HiSoC base models, applied to the PRE-CVT versus NON-CVT task, demonstrated relatively low Matthews correlation coefficients (MCC = 0.30 and 0.38, respectively), yet their performance weakly exceeded that of null models (p = 0.057 and p = 0.06, respectively). In contrast, the CAARMS-based model achieved the same MCC as the HiSoC model (MCC = 0.38) but failed to outperform the null models. This suggests that the number of variables influences a model’s ability to surpass null expectations, as the CAARMS model included seven variables while HiSoC had only three. Such an effect aligns with expectations that models with more variables can capture greater complexity but possibly at the cost of overfitting, which inflates apparent performance. Moreover, nearly all variable sets yielded MCC values above 0.4, indicating better-than-random classification, yet only three models (BACS, HiSoC, and CAARMS + RISK + HiSoC) significantly outperformed null models. These findings underscore that strong classification metrics alone may not reflect meaningful pattern recognition, highlighting the importance of rigorous validation against null distributions to evaluate true model utility.

Interestingly, while models based individually on BACS and HiSoC variables outperformed null models, the combined model incorporating CAARMS, HiSoC, and BACS variables did not (p = 0.72). This may be attributed to a moderate correlation between HiSoC and BACS variables (r = 0.402), which likely resulted in limited incremental information when combined. Consequently, the added redundancy constrained the combined model’s ability to outperform null models despite comparable dimensionality.

The identification of HiSoC features as particularly information-rich is consistent with our recent findings demonstrating that sentiment features derived from the HiSoC task strongly predict UHR status [44]. These HiSoC-derived sentiment features outperformed a range of other natural language processing features in predictive accuracy. This alignment reinforces the value of the HiSoC modality as a key contributor to effective UHR classification models and highlights the potential of sentiment analysis of social challenge tasks as a biomarker in psychosis risk prediction. In practical use, capturing and incorporating such linguistic and social cognition signals can enhance the sensitivity and specificity of predictive algorithms aimed at early detection and intervention in psychosis.

### Impact

Our approach for systematically evaluating prediction models is straightforward, scalable, and effective. Although our findings are exploratory, given the study’s application to a limited, single-site dataset, extending this analysis to include multiple cohorts could significantly enhance our understanding of the information value of different data modalities. This would not only promote the targeted collection of meaningful variables but also advance model validation and evaluation processes by addressing challenges related to generalizability and real-world clinical applicability.

Importantly, we observed that models failing to outperform null models can still exhibit seemingly good classification metrics, underscoring the need for an additional evaluation layer in model development studies. High classification performance alone is insufficient if it can be easily matched by chance — a model with moderate performance that consistently surpasses null models may hold greater translational promise. Therefore, we emphasize the critical role of rigorous validation standards to guide decisions on which models warrant further development or clinical deployment. Adopting such formal evaluation principles will help prioritize models with true predictive utility, balancing statistical robustness with practical considerations in mental health prognostics.

### Limitations

Our study has several limitations that temper the generalizability of the findings. First, our sample size is relatively small, comprising 56 UHR individuals, while the largest model evaluated included 21 variables. Such a low sample-to-variable ratio increases the risk of overfitting, though this is mitigated by the study’s focus on assessing the marginal utility of individual modalities rather than developing fully generalizable models. Second, more advanced PRS construction methods such as PRS-CS [45], which employ Bayesian shrinkage priors to better account for linkage disequilibrium, may yield improved polygenic scores compared to the clumping-and-thresholding approach used here. However, given the limited predictive contribution of PRS observed in the current study and the small target sample size, the choice of PRS construction method is unlikely to materially alter the conclusions. Future studies with larger cohorts should evaluate whether such methodological advances can enhance multimodal prediction of psychosis outcomes in East Asian populations. Third, we lacked access to external cohorts for independent validation, limiting demonstration of our models’ reproducibility across sites. Forth, our internal validation employed 1,000 bootstrap iterations, which may yield unstable performance estimates. However, computational constraints arose from the extensive model fitting required—over 1.9 billion models—to generate null distributions and assess significance, making higher iteration counts infeasible. Future method development could focus on more efficient empirical performance estimation to enable scalability. Lastly, while our evaluation considered only the types of assessments performed, differences in the time and resources required for each assessment were not accounted for. Understanding these logistical costs is essential for assessing clinical feasibility and should be a priority in future research.

## Supporting information

S1 Appendix Supplementary methods for polygenic risk score and RNA-seq processing and Tables A–K. (DOCX)

S1 Fig Sensitivity analysis comparing single imputation (m = 1, blue) with multiple imputation (m = 10, red) for selected models. Error bars represent the range across 10 independent imputations. Missing data were concentrated in HiSoC variables (35–37% in UHR). Models without imputed variables (CAARMS) show near-identical performance, while the best-performing model (CAARMS + RISK + HiSoC) remains stable across imputations for both prediction tasks. (TIF)
