# Supplementary material for: Dissecting multimodal predictors of psychosis risk and remission in youth at ultra‑high risk of developing psychosis: An exploratory study
Source: PLOS Ment Health. 2026 Aug 21;3(8):e0000686. doi: 10.1371/journal.pmen.0000686 (PMC13496409; doi:10.1371/journal.pmen.0000686)
Supplement: Supplementary file 1 [file pmen.0000686.s001.docx]

**Dissecting Multimodal Predictors of Psychosis Risk and Remission in Youth at Ultra‑High Risk of Developing Psychosis**

**S1 Appendix**

Weijia Kong^1,^^, Samuel Ming Xuan Tan^1,2^, Matheus Calvin Lokadjaja^1^, Jordon Junyang Kho^1^, Zixu Yang^3^, Jieyin Yee^3^, Max Lam^1,3^, Jimmy Lee^1,3*^, Wilson Wen Bin Goh^1,2,3,4,5,6,^*^

1. Lee Kong Chian School of Medicine, Nanyang Technological University, Singapore
2. School of Biological Sciences, Nanyang Technological University, Singapore
3. Institute of Mental Health, Singapore
4. Centre for Biomedical Informatics, Nanyang Technological University, Singapore
5. Centre of AI in Medicine, Nanyang Technological University, Singapore
6. Division of Neurology, Department of Brain Sciences, Faculty of Medicine, Imperial College London

^ First Author(s)

* Corresponding Author(s): Jimmy Lee, jimmy.lee@nhghealth.com.sg; Wilson Wen Bin Goh, wilsongoh@ntu.edu.sg

**Processing of PRS scores**

Blood samples collected from the participants were genotyped using the Illumina OmniZhongHua-8 BeadChip. Standard quality control procedures were performed in PLINK 1.9 (Purcell et al., 2007) – single nucleotide polymorphisms (SNPs) with minor allele frequency (MAF) < 0.01, call rate < 0.98, Hardy-Weinberg equilibrium p-value < 1e-06; and exclude individuals with mismatch between recorded and genotyped sex, and related individuals. Cryptic relatedness was identified with identity by descent method (pi-hat > 0.2) (please see (Lim et al., 2020) for additional processing details).

Imputation was conducted separately for each ancestry group using Minimac3 (MaCH) (Das et al., 2016) against the full 1000 Genomes phase 3 reference panel (Consortium and others, 2012). The imputed SNPs underwent a second round of quality control using the same criteria as before, with an additional filter for imputation quality score > 0.9. The final imputed data were merged on shared SNPs for comprehensive sample analysis, yielding a total of 3,349,959 high-quality SNPs.

Polygenic risk score of each individual was calculated as the weighted sum of risk alleles using GWAS summary statistics from the PGC East-Asian schizophrenia meta-analysis (PGC SCZ-EAS) (Lam et al., 2019) as discovery samples. PRS were calculated across ten p-value thresholds (ρ_T_ ≤ 5 x 10−08, 1 x 10−05, 1 x 10−04, 1 x 10−03, 0.01, 0.05, 0.1, 0.2, 0.5, 1), and standardized using the means and standard deviations from healthy controls. The PRS were generated using PRSice-2 (Choi and O’Reilly, 2019). For this study, we selected the PRS calculated at the ρ_T_ that achieved the highest Nagelkerke R^2^. This was found to be the PRS calculated at ρ_T_ = 0.2 with Nagelkerke R^2^ = 0.081.

**RNA-seq data sequencing and processing**

Venous whole blood samples were collected from all participants into Tempus™ Blood RNA tubes (Thermo Fisher Scientific) and stored at −80°C. Total RNA extraction was carried out using the Tempus™ Spin RNA Isolation Kit (Thermo Fisher Scientific) following the manufacturer’s guidelines. The quality and concentration of the extracted RNA were assessed using the 2100 Bioanalyzer System (Agilent). Library preparation and sequencing were performed on eight Illumina NovaSeq lanes (2x151bp). The quality of the sequencing data was evaluated with FastQC v0.11.9 (Schmieder and Edwards, 2011). Sequences were aligned to the Ensembl GRCh38 reference genome using STAR 2.7.9a (Dobin et al., 2013). The aligned reads were quantified using the featureCount function from the Subread 2.0.3 R Package (Liao et al., 2019). Genes with zero variance and a mean count of less than 1 were excluded, resulting in a total of 21,810 genes for analysis. All data processing and analysis were conducted using R-4.0.3 (R Core Team, 2013).

We next conducted a power analysis using the ssizeRNA R package (Bi and Liu, 2016). The criteria were set at an absolute log2 fold-change greater than 1 and a false discovery rate of 0.05. Based on the low number of differentially expressed genes reported in similar studies (Chaumette et al., 2019; Mongan et al., 2020), we estimated the expected proportion of differentially expressed genes at 0.005. Our analysis indicated that a sample size of 11 would provide a statistical power of 0.81. With 13 converter samples, our study achieved a power of 0.851.

**Selection of RNA-seq variables**

26 candidate RNA-seq variables were selected from first compiling a list of candidate biomarkers using published literature of cross-sectional studies involving treatment naïve participants with first episode psychosis and longitudinal study involving samples collected from UHR individuals before and after FEP namely (Chaumette et al., 2019; Gouvea et al., 2016; Kurian et al., 2011; Kuzman et al., 2009; Olincy et al., 2011; Wagh et al., 2021) The list of genes from each study is collated in Table A.

Using the RNA-seq data from the LYRIKS cohort, we calculated the Cohen’s D between samples collected before psychosis conversion and samples collected after psychosis conversion for all 26 candidate genes (Table B).

The Cohen’s D is a measure of effect size and is calculated as:

$$D=\frac{\bar{x}_{1}{-\bar{x}}_{2}}{s}$$

Where $\bar{x}_{1}$ and $\bar{x}_{2}$ are the sample means of group 1 and group 2 respectively. $s$ is the pooled standard deviation defined as

$s=\sqrt{\frac{\left( n_{1}-1 \right)s_{1}^{2}+\left( n_{2}-1 \right)s_{2}^{2}}{n_{1}+n_{2}-2}}$

Where $n_{1}$, $s_{1}$ and $n_{2},s_{2}$ are the sample size and standard deviated for groups 1 and 2 respectively.

The larger the Cohen’s D is, the stronger the association of the variable with psychosis conversion. We selected the top 5 genes based on Cohen’s D was selected to represent genes associated with psychosis conversion.

We next calculated Cohen’s D for each gene for their effect size in psychosis conversion. We selected the top 5 (average number of variables across the other modalities) genes namely CCDC134; BRCA1; EGR1; NLN; and TOX for subsequent analysis.

**Table A. Candidate RNA biomarkers**

| Gene | Ensembl ID | Article |
| --- | --- | --- |
| Mpp3 | ENSG00000161647 | (Kurian et al., 2011) |
| Phlda1 | ENSG00000139289 |  |
| S100a6 | ENSG00000197956 |  |
| Drd2 | ENSG00000149295 |  |
| Egr1 | ENSG00000120738 |  |
| Nrg1 | ENSG00000157168 |  |
| Fn1 | ENSG00000115414 |  |
| Tob2 | ENSG00000183864 |  |
| Gpm6b | ENSG00000046653 |  |
| *MBP* | ENSG00000197971 | (Gouvea et al., 2016; Ota et al., 2015) |
| *NDEL1* | ENSG00000166579 |  |
| CPT1A | ENSG00000110090 | (Chaumette et al., 2019) |
| GSTM5 | ENSG00000134201 |  |
| NRP1 | ENSG00000099250 |  |
| UBD | ENSG00000213886 | (Wagh et al., 2021) |
| BRCA1 | ENSG00000012048 |  |
| AKT1 | ENSG00000142208 |  |
| CCDC134 | ENSG00000100147 |  |
| ZIC2 | ENSG00000043355 |  |
| TOX | ENSG00000198846 |  |
| EIF2D | ENSG00000143486 |  |
| SLC2A3 | ENSG00000059804 | (Kuzman et al., 2009) |
| DAAM2 | ENSG00000146122 |  |
| myosin C | ENSG00000197879 |  |
| NLN | ENSG00000123213 |  |
| DISC1 | ENSG00000162946 | (Olincy et al., 2011) |

**Table B. Effect size of candidate genes in LYRIKS data**

| Ensembl ID | Gene Symbol | Cohen’s D |
| --- | --- | --- |
| ENSG00000100147 | CCDC134 | 0.915156 |
| ENSG00000012048 | BRCA1 | 0.693254 |
| ENSG00000120738 | EGR1 | 0.629875 |
| ENSG00000123213 | NLN | 0.565769 |
| ENSG00000198846 | TOX | 0.512279 |
| ENSG00000166579 | NDEL1 | -0.4964 |
| ENSG00000146122 | DAAM2 | -0.47259 |
| ENSG00000197879 | MYO1C | 0.425774 |
| ENSG00000099250 | NRP1 | 0.399192 |
| ENSG00000183864 | TOB2 | 0.370598 |
| ENSG00000197956 | S100A6 | -0.3655 |
| ENSG00000115414 | FN1 | 0.296246 |
| ENSG00000110090 | CPT1A | -0.27708 |
| ENSG00000143486 | EIF2D | -0.20351 |
| ENSG00000046653 | GPM6B | 0.201488 |
| ENSG00000059804 | SLC2A3 | -0.19859 |
| ENSG00000139289 | PHLDA1 | 0.162383 |
| ENSG00000142208 | AKT1 | -0.15385 |
| ENSG00000161647 | MPP3 | -0.1089 |
| ENSG00000162946 | DISC1 | -0.01852 |
| ENSG00000157168 | NRG1 | -0.01508 |
| ENSG00000197971 | MBP | -0.01288 |

**Table C. Data abbreviation and type**

| Variable | Abbreviation | Data type |
| --- | --- | --- |
| **CAARMS** |  |  |
| Genetic vulnerability | Caarms_vul | Binary |
| Attenuated psychosis syndrome (subgroup 2a) | Caarms_aps2a | Binary |
| Attenuated psychosis syndrome (subgroup 2b) | Caarms_aps2b | Binary |
| Brief limited intermittent psychosis | Caarms_blips | Binary |
| Distress – Unusual thought content | UTC_distress | Continuous |
| Distress – Non-bizarre ideas | NBI distress | Continuous |
| Distress – perceptual abnormalities | PA distress | Continuous |
| Distress – disorganized speech | DS distress | Continuous |
| **BACS** |  |  |
| Verbal Memory | bacs_vm | Continuous |
| Digit Sequencing | bacs_ds | Continuous |
| Token Motor Task | bacs_tmt | Continuous |
| Verbal Fluency | bacs_vf | Continuous |
| Symbol Coding | bacs_sc | Continuous |
| Tower of London | bacs_tol | Continuous |
| **HiSoC** |  |  |
| Affect | hisoc_affect | Continuous |
| Odd behaviour and speech | hisoc_odd | Continuous |
| Social-interpersonal | hisoc_social | Continuous |
| **RISK** |  |  |
| History of depressive disorder | Depression_lp | Binary |
| Family history of depressive and/or anxiety disorder | Fam_dep_anx | Binary |
| Family history of psychosis | Fam_scz_psy | Binary |
| Maternal age at birth | Maternal_age | Continuous |
| Paternal age at birth | Paternal_age | Continuous |
| Low education attainment relative to age | Low_edu_rel_age | Binary |
| Late graduation from primary education | Late_psle | Binary |
| Childhood or adolescent smoker | child_adoles_smoker | Binary |
| **Polygenic Risk Score** |  |  |
| Polygenic Risk Score | PRS | Continuous |
| **RNA markers** |  |  |
| CCDC134 | CCDC134 | Continuous |
| BRCA1 | BRCA1 | Continuous |
| EGR1 | EGR1 | Continuous |
| NLN | NLN | Continuous |
| TOX | TOX | Continuous |

**Table D. Models and number of continuous and binary variables**

| Model | Number of Continuous variables | Number of binary variables |
| --- | --- | --- |
| Base models |  |  |
| CAARMS | 4 | 4 |
| RISK | 2 | 6 |
| HiSoC | 3 | 0 |
| BACS | 5 | 0 |
| RNA | 5 | 0 |
| CAARMS + 1 modality |  |  |
| CAARMS + RISK | 2 | 6 |
| CAARMS + HiSoC | 3 | 0 |
| CAARMS + BACS | 5 | 0 |
| CAARMS + RNA | 5 | 0 |
| CAARMS + PRS | 1 | 0 |
| CAARMS + 2 modality |  |  |
| CAARMS + RISK + HiSoC | 5 | 6 |
| CAARMS + RISK + BACS | 7 | 6 |
| CAARMS + RISK + RNA | 7 | 6 |
| CAARMS + RISK + PRS | 3 | 6 |
| CAARMS + HiSoC + BACS | 8 | 0 |
| CAARMS + HiSoC + RNA | 8 | 0 |
| CAARMS + HiSoC + PRS | 4 | 0 |
| CAARMS + BACS + RNA | 10 | 0 |
| CAARMS + BACS + PRS | 6 | 0 |
| CAARMS + RNA + PRS | 6 | 0 |

**Table E. Performance metrics and empirical p-value of base models**

| Task | Variable sets | MCC [p] | SP [p] | SE [p] |
| --- | --- | --- | --- | --- |
| Pre-Conversion vs non-conversion | CAARMS | 0.38 [0.276] | 0.567 [0.575] | 0.794 [0.507] |
|  | RISK | 0.365 [0.313] | 0.547 [0.586] | 0.794 [0.524] |
|  | HiSoC | 0.304 [0.0566] | 0.538 [0.477] | 0.158 [0.975] |
|  | BACS | 0.378 [0.0596] | 0.716 [0.196] | 0.719 [0.485] |
|  | RNA | 0.106 [0.948 | 0.444 [0.731] | 0.654 [0.623] |
| Pre-remission vs non-remission | CAARMS | 0.275 [0.5] | 0.289 [0.483] | 0.621 [0.483] |
|  | RISK | 0.312 [0.328] | 0.722 [0.3] | 0.335 [0.3] |
|  | HiSoC | 0.153 [0.414] | 0.473 [0.414] | 0.147 [0.414] |
|  | BACS | 0.121 [0.845] | 0.339 [0.908] | 0.555 [0.908] |
|  | RNA | 0.197 [0.49] | 0.745 [0.4904] | 0.323 [0.490] |

**Table F. Coefficients of base models**

|  | pre-cvt vs non-cvt task | | | | | pre-remission vs non-remission task | | | | |
| --- | --- | --- | --- | --- | --- | --- | --- | --- | --- | --- |
|  | CAARMS | BACS | HiSoC | RISK | RNA | CAARMS | BACS | HiSoC | RISK | RNA |
| caarms_vul | -0.40495 | - | - | - | - | 0.481634 | - | - | - | - |
| caarms_aps2a | -0.36672 | - | - | - | - | 0.218716 | - | - | - | - |
| caarms_aps2b | 0* | 0* | 0* | 0* | 0* | 0* | 0* | 0* | 0* | 0* |
| caarms_blips | -3.8427 | - | - | - | - | -4.2826 | - | - | - | - |
| caarms_utc_distress | -0.01061 | - | - | - | - | -0.02483 | - | - | - | - |
| caarms_nbi_distress | 0.025312 | - | - | - | - | 0.002925 | - | - | - | - |
| caarms_pa_distress | 0.016999 | - | - | - | - | -0.00257 | - | - | - | - |
| caarms_ds_distress | 0.005643 | - | - | - | - | 0.027642 | - | - | - | - |
| bacs_vm | - | 0.019754 | - | - | - | - | 0.03298 | - | - | - |
| bacs_ds | - | 0.057176 | - | - | - | - | -0.03446 | - | - | - |
| bacs_tmt | - | -0.02375 | - | - | - | - | 0.02989 | - | - | - |
| bacs_vf | - | -0.0789 | - | - | - | - | -0.03512 | - | - | - |
| bacs_sc | - | -0.03873 | - | - | - | - | 0.009853 | - | - | - |
| bacs_tol | - | -0.17033 | - | - | - | - | -0.28917 | - | - | - |
| hisoc_affect | - | - | -0.26898 | - | - | - | - | -0.08164 | - | - |
| hisoc_odds | - | - | -0.17012 | - | - | - | - | 0.169193 | - | - |
| hisoc_social | - | - | 0.134802 | - | - | - | - | -0.30677 | - | - |
| depression_lp | - | - | - | -1.19049 | - | - | - | - | -0.46937 | - |
| fam_dep_anx | - | - | - | -1.19546 | - | - | - | - | 1.635346 | - |
| fam_scz_psy | - | - | - | -0.74788 | - | - | - | - | -0.61786 | - |
| maternal_age_at_birth | - | - | - | 0.166792 | - | - | - | - | 0.155389 | - |
| paternal_age_at_birth | - | - | - | -0.13222 | - | - | - | - | -0.10456 | - |
| low_edu_rel_age | - | - | - | 0.60925 | - | - | - | - | 0.335162 | - |
| late_psle | - | - | - | 2.456053 | - | - | - | - | 2.918939 | - |
| child_adoles_smoker | - | - | - | 1.012337 | - | - | - | - | -0.83408 | - |
| ENSG00000260070 | - | - | - | - | 1.226422 | - | - | - | - | 0.340042 |
| ENSG00000253005 | - | - | - | - | 0.24076 | - | - | - | - | 0.538743 |
| ENSG00000237757 | - | - | - | - | 0.934202 | - | - | - | - | 0.522135 |
| ENSG00000277692 | - | - | - | - | 0.293599 | - | - | - | - | -0.21543 |
| ENSG00000273437 | - | - | - | - | -0.27384 | - | - | - | - | 0.065923 |

*Zero-variance feature; no participant in the analytic sample met this intake criterion

**Table G. Performance metrics and empirical p-value of CAARMS + 1 modality models**

| Task | Variable sets | MCC [p] | SP [p] | SE [p] |
| --- | --- | --- | --- | --- |
| Pre-Conversion vs non-conversion | CAARMS + RISK | 0.644 [0.308] | 0.839 [0.225] | 0.833 [0.729] |
|  | CAARMS + HiSoC | 0.518 [0.246] | 0.768 [0.164] | 0.78 [0.845] |
|  | CAARMS + BACS | 0.619 [0.141] | 0.819 [0.143] | 0.842 [0.659] |
|  | CAARMS + RNA | 0.495 [0.842] | 0.64 [0.819] | 0.9 [0.376] |
|  | CAARMS + PRS | 0.414 [0.385] | 0.591 [0.434] | 0.786 [0.897] |
| Pre-remission vs non-remission | CAARMS + RISK | 0.571 [0.215] | 0.781 [0.313] | 0.737 [0.719] |
|  | CAARMS + HiSoC | 0.383 [1] | 0.721 [0.283] | 0.658 [0.990] |
|  | CAARMS + BACS | 0.381 [0.797] | 0.375 [0.958] | 0.665 [0.851] |
|  | CAARMS + RNA | 0.347 [0.967] | 0.552 [0.734] | 0.791 [0.485] |
|  | CAARMS + PRS | 0.276 [0.951] | 0.351 [0.321] | 0.614 [0.978] |

**Table H. Coefficients of CAARMS + 1 modality models**

|  | pre-cvt vs non-cvt task | | | | | pre-remission vs non-remission task | | | | |
| --- | --- | --- | --- | --- | --- | --- | --- | --- | --- | --- |
|  | CAARMS + RISK | CAARMS + HiSoC | CAARMS + BACS | CAARMS + RNA | CAARMS + PRS | CAARMS + RISK | CAARMS + HiSoC | CAARMS + BACS | CAARMS + RNA | CAARMS + PRS |
| caarms_vul | -3.69248 | -0.26715 | -4.14017 | 0.041548 | -0.45105 | 3.034997 | 1.221694 | 0.45912 | 1.363362 | 0.309131 |
| caarms_aps2a | 1.144332 | 1.105922 | -1.01727 | -0.68502 | -0.77128 | 0.70813 | 0.774736 | 0.488787 | 0.183274 | 0.135824 |
| caarms_aps2b | 0* | 0* | 0* | 0* | 0* | 0* | 0* | 0* | 0* | 0* |
| caarms_blips | -12.8457 | -3.35404 | -3.52543 | -3.11424 | -5.13602 | -2.62936 | -1.81979 | -4.33482 | -4.86389 | -4.1813 |
| caarms_utc_distress | -0.07107 | -0.02001 | 0.013132 | -0.00263 | -0.02097 | -0.02428 | -0.02941 | -0.01844 | -0.0265 | -0.02279 |
| caarms_nbi_distress | 0.112316 | 0.011041 | 0.032436 | 0.034512 | 0.032995 | 0.011436 | 0.004817 | -0.00187 | 0.000317 | -0.00072 |
| caarms_pa_distress | 0.032731 | 0.016977 | 0.017267 | 0.019251 | 0.017173 | -0.01099 | -0.00848 | -0.01073 | -0.00265 | -7.35E-05 |
| caarms_ds_distress | 0.048188 | 0.01462 | -0.04371 | -0.00807 | 0.012421 | -0.00356 | 0.000335 | 0.029153 | 0.039984 | 0.023039 |
| bacs_vm | - | - | 0.03638 | - | - | - | - | 0.047033 | - | - |
| bacs_ds | - | - | -0.12507 | - | - | - | - | -0.08389 | - | - |
| bacs_tmt | - | - | 0.160789 | - | - | - | - | 0.027068 | - | - |
| bacs_vf | - | - | -0.21364 | - | - | - | - | -0.02123 | - | - |
| bacs_sc | - | - | -0.07257 | - | - | - | - | -0.00894 | - | - |
| bacs_tol | - | - | -0.27561 | - | - | - | - | -0.39383 | - | - |
| hisoc_affect | - | -0.20336 | - | - | - | - | -0.05731 | - | - | - |
| hisoc_odds | - | -0.32866 | - | - | - | - | 0.177135 | - | - | - |
| hisoc_social | - | 0.268537 | - | - | - | - | -0.41953 | - | - | - |
| depression_lp | -0.3246 | - | - | - | - | -1.06082 | - | - | - | - |
| fam_dep_anx | -1.9025 | - | - | - | - | 2.695437 | - | - | - | - |
| fam_scz_psy | 1.541807 | - | - | - | - | -2.175 | - | - | - | - |
| maternal_age_at_birth | 0.098141 | - | - | - | - | 0.174954 | - | - | - | - |
| paternal_age_at_birth | -0.05349 | - | - | - | - | -0.12393 | - | - | - | - |
| low_edu_rel_age | 2.519341 | - | - | - | - | 0.930817 | - | - | - | - |
| late_psle | 8.972075 | - | - | - | - | 4.535102 | - | - | - | - |
| child_adoles_smoker | 1.115062 | - | - | - | - | 0.586683 | - | - | - | - |
| ENSG00000260070 | - | - | - | 1.280475 | - | - | - | - | 0.671215 | - |
| ENSG00000253005 | - | - | - | -0.24549 | - | - | - | - | 0.260233 | - |
| ENSG00000237757 | - | - | - | 0.648933 | - | - | - | - | -0.22187 | - |
| ENSG00000277692 | - | - | - | -0.05793 | - | - | - | - | -0.62806 | - |
| ENSG00000273437 | - | - | - | 0.407495 | - | - | - | - | 0.330885 | - |
| prs | - | - | - | - | -0.35223 | - | - | - | - | 0.065433 |

*Zero-variance feature; no participant in the analytic sample met this intake criterion.

**Table I. Performance metrics and empirical p-value of CAARMS + 2 modality models**

| Task | Variable sets | MCC [p] | SP [p] | SE [p] |
| --- | --- | --- | --- | --- |
| Pre-Conversion vs non-conversion | CAARMS + RISK + HiSoC | 0.71 [0.055] | 0.93 [0.0278] | 0.783 [0.883] |
|  | CAARMS + RISK + BACS | 0.66 [0.413] | 0.888 [0.132] | 0.782 [0.739] |
|  | CAARMS + RISK + RNA | 0.644 [0.557] | 0.881 [0.151] | 0.781 [0.743] |
|  | CAARMS + RISK + PRS | 0.644 [0.383] | 0.881 [0.128] | 0.781 [0.934] |
|  | CAARMS + HiSoC + BACS | 0.652 [0.277] | 0.652 [0.276] | 0.835 [0.256] |
|  | CAARMS + HiSoC + RNA | 0.55 [0.928] | 0.55 [0.928] | 0.809 [0.373] |
|  | CAARMS + HiSoC +PRS | 0.519 [0.443] | 0.519 [0.442] | 0.763 [0.245] |
|  | CAARMS + BACS + RNA | 0.633 [0.545] | 0.633 [0.545 | 0.779 [0.685] |
|  | CAARMS + BACSs + PRS | 0.633 [0.182] | 0.635 [0.182] | 0.833 [0.146] |
|  | CAARMS + RNA + PRS | 0.532 [0.798] | 0.532 [0.798] | 0.66 [0.84] |
| Pre-remission vs non-remission | CAARMS + RISK + HiSoC | 0.642 [0.233] | 0.818 [0.342] | 0.77 [0.742] |
|  | CAARMS + RISK + BACS | 0.632 [0.493] | 0.784 [0.585] | 0.822 [0.559] |
|  | CAARMS + RISK + RNA | 0.632 [0.486] | 0.718 [0.819] | 0.889 [0.23] |
|  | CAARMS + RISK + PRS | 0.661 [0.104] | 0.727 [0.568] | 0.815 [0.481] |
|  | CAARMS + HiSoC + BACS | 0.46 [0.853] | 0.716 [0.523] | 0.726 [0.765] |
|  | CAARMS + HiSoC + RNA | 0.456 [0.865] | 0.735 [0.454] | 0.673 [0.896] |
|  | CAARMS + HiSoC +PRS | 0.426 [0.263] | 0.717 [0.203] | 0.705 [0.776] |
|  | CAARMS + BACS + RNA | 0.534 [0.779] | 0.541 [0.980] | 0.812 [0.522] |
|  | CAARMS + BACSs + PRS | 0.414 [0.754] | 0.581 [0.762] | 0.703 [0.784] |
|  | CAARMS + RNA + PRS | 0.38 [0.943] | 0.423 [0.963] | 0.78 [0.543] |

**Table J. Coefficients of CAARMS + 2 modality models (pre-cvt vs non-cvt task)**

|  | CAARMS + RISK + HiSoC | CAARMS + RISK + BACS | CAARMS + RISK + RNA | CAARMS + RISK + PRS | CAARMS + HiSoC + BACS | CAARMS + HiSoC + RNA | CAARMS + HiSoC + PRS | CAARMS + BACS + RNA | CAARMS + BACS + PRS | CAARMS + RNA + PRS |
| --- | --- | --- | --- | --- | --- | --- | --- | --- | --- | --- |
| caarms_vul | -5.03066 | -9.0815 | -0.89014 | 2.108139 | -1.5248 | -0.32055 | 0.08943 | -4.37776 | -5.66125 | 0.211198 |
| caarms_aps2a | 4.257355 | -2.41267 | 3.390337 | 5.962133 | 0.63832 | 2.478924 | 0.563437 | -3.1336 | -1.5784 | -0.60038 |
| caarms_aps2b | 0* | 0* | 0* | 0* | 0* | 0* | 0* | 0* | 0* | 0* |
| caarms_blips | -4.13799 | -14.1847 | -1.89982 | -9.64123 | -1.0728 | -1.20042 | -1.47877 | -6.59322 | -4.94627 | -2.66942 |
| caarms_utc_distress | -0.11355 | -0.00688 | -0.03504 | -0.07598 | -0.02073 | 0.00088 | -0.01318 | 0.008235 | 0.028208 | -0.00342 |
| caarms_nbi_distress | 0.012006 | -0.02907 | 0.110696 | 0.132325 | -0.00357 | 0.009429 | 0.017834 | 0.04972 | 0.051856 | 0.014199 |
| caarms_pa_distress | 0.091502 | 0.105249 | 0.11983 | 0.025777 | 0.020154 | 0.041701 | 0.012884 | 0.016579 | 0.018987 | 0.023778 |
| caarms_ds_distress | -0.05884 | 0.001401 | 0.018388 | 0.01443 | -0.02862 | -0.01931 | -0.00052 | 0.01836 | -0.11166 | -0.00221 |
| bacs_vm | - | -0.08426 | - | - | -0.06504 | - | - | 0.032047 | -0.00093 | - |
| bacs_ds | - | 0.000298 | - | - | 0.065388 | - | - | 0.130713 | -0.04418 | - |
| bacs_tmt | - | -0.04826 | - | - | -0.06543 | - | - | 0.204124 | 0.253402 | - |
| bacs_vf | - | -0.18712 | - | - | -0.20787 | - | - | -0.21826 | -0.28237 | - |
| bacs_sc | - | -0.18942 | - | - | 0.009973 | - | - | -0.0464 | -0.07864 | - |
| bacs_tol | - | 0.04057 | - | - | 0.041117 | - | - | -1.19786 | -0.53565 | - |
| hisoc_affect | 0.254103 | - | - | - | -0.39469 | -0.22616 | -0.15705 | - | - | - |
| hisoc_odds | -1.50959 | - | - | - | -0.18707 | -0.69389 | -0.26743 | - | - | - |
| hisoc_social | -1.48985 | - | - | - | 0.145574 | 0.289564 | 0.236642 | - | - | - |
| depression_lp | 1.899013 | -1.53993 | 2.737576 | -3.03772 | - | - | - | - | - | - |
| fam_dep_anx | -4.16348 | -1.51994 | 0.166448 | 0.378902 | - | - | - | - | - | - |
| fam_scz_psy | 8.058984 | 2.706536 | 4.616923 | -1.9518 | - | - | - | - | - | - |
| maternal_age_at_birth | 0.775377 | 0.363466 | 0.037255 | 0.283765 | - | - | - | - | - | - |
| paternal_age_at_birth | -0.49852 | -0.13461 | 0.148144 | -0.3162 | - | - | - | - | - | - |
| low_edu_rel_age | -4.7967 | 2.577354 | 6.392266 | 5.275503 | - | - | - | - | - | - |
| late_psle | 10.67384 | 2.900705 | 11.3497 | 11.60753 | - | - | - | - | - | - |
| child_adoles_smoker | 2.873541 | -0.2505 | 0.469712 | 3.368746 | - | - | - | - | - | - |
| ENSG00000260070 | - | - | 3.556609 | - | - | 0.948237 | - | 1.242375 | - | 0.313926 |
| ENSG00000253005 | - | - | 3.59305 | - | - | -1.63272 | - | -1.20716 | - | -0.07329 |
| ENSG00000237757 | - | - | 1.241497 | - | - | 1.061767 | - | -0.313 | - | 0.939086 |
| ENSG00000277692 | - | - | 4.141443 | - | - | 0.965897 | - | 0.916394 | - | 0.696891 |
| ENSG00000273437 | - | - | -1.56226 | - | - | 1.222654 | - | 0.145817 | - | 0.068576 |
| prs | - | - | - | 2.518101 | - | - | 0.09798 | - | 0.341617 | 0.185616 |

*Zero-variance feature; no participant in the analytic sample met this intake criterion.

**Table K. Coefficients of CAARMS + 2 modality models (pre-remission vs non-remission task)**

|  | CAARMS + RISK + HiSoC | CAARMS + RISK + BACS | CAARMS + RISK + RNA | CAARMS + RISK + PRS | CAARMS + HiSoC + BACS | CAARMS + HiSoC + RNA | CAARMS + HiSoC + PRS | CAARMS + BACS + RNA | CAARMS + BACS + PRS | CAARMS + RNA + PRS |
| --- | --- | --- | --- | --- | --- | --- | --- | --- | --- | --- |
| caarms_vul | 3.013819 | 3.31357 | 4.531823 | 3.645698 | 1.322307 | 1.192576 | 1.338142 | 0.77028 | -0.46939 | 0.815093 |
| caarms_aps2a | 1.923746 | 0.764079 | 3.714631 | 0.64104 | 1.591231 | 0.715569 | 0.369199 | 0.685912 | -0.24304 | -0.76858 |
| caarms_aps2b | 0* | 0* | 0* | 0* | 0* | 0* | 0* | 0* | 0* | 0* |
| caarms_blips | -2.22967 | -2.98694 | -4.39282 | -3.87586 | 0 | -2.04791 | 0 | -3.87619 | -5.43858 | -3.32459 |
| caarms_utc_distress | -0.03358 | -0.02654 | -0.03772 | -0.01774 | -0.02799 | -0.03014 | -0.0229 | -0.02531 | -0.01681 | -0.01752 |
| caarms_nbi_distress | 0.007297 | 0.014138 | 0.003653 | 0.020292 | 0.000464 | 0.005936 | 0.004915 | -0.00324 | 0.00191 | -0.00081 |
| caarms_pa_distress | -0.01746 | -0.0095 | -0.02179 | -0.00891 | -0.01549 | -0.01015 | -0.00447 | -0.01804 | -0.00291 | -0.0053 |
| caarms_ds_distress | 0.004996 | 0.005812 | 0.0495 | 0.0178 | -0.02296 | -0.00217 | -0.01844 | 0.027362 | 0.027031 | 0.020533 |
| bacs_vm | - | 0.064611 | - | - | 0.108301 | - | - | 0.098443 | -0.00802 | - |
| bacs_ds | - | -0.00098 | - | - | -0.00177 | - | - | -0.03452 | -0.09493 | - |
| bacs_tmt | - | 0.003903 | - | - | 0.032552 | - | - | 0.011354 | 0.066886 | - |
| bacs_vf | - | -0.02345 | - | - | -0.02692 | - | - | -0.03474 | -0.04179 | - |
| bacs_sc | - | -0.02306 | - | - | -0.04093 | - | - | -0.04553 | 0.007281 | - |
| bacs_tol | - | -0.07593 | - | - | -0.52469 | - | - | -0.34275 | -0.30843 | - |
| hisoc_affect | -0.09509 | - | - | - | -0.09505 | -0.10123 | -0.11858 | - | - | - |
| hisoc_odds | 0.068248 | - | - | - | -0.06911 | 0.017255 | 0.071069 | - | - | - |
| hisoc_social | -0.40852 | - | - | - | -0.32044 | -0.17564 | -0.224 | - | - | - |
| depression_lp | -1.10543 | -0.94319 | -1.46113 | -0.32868 | - | - | - | - | - | - |
| fam_dep_anx | 2.51555 | 3.059694 | 3.365628 | 2.878596 | - | - | - | - | - | - |
| fam_scz_psy | -0.85665 | -1.64791 | -2.53157 | -2.67336 | - | - | - | - | - | - |
| maternal_age_at_birth | 0.108933 | 0.168798 | 0.147368 | 0.162557 | - | - | - | - | - | - |
| paternal_age_at_birth | -0.02284 | -0.11018 | -0.03173 | -0.13721 | - | - | - | - | - | - |
| low_edu_rel_age | 0.808427 | 0.796922 | 1.999942 | 2.164265 | - | - | - | - | - | - |
| late_psle | 3.766755 | 3.818915 | 3.615682 | 4.572592 | - | - | - | - | - | - |
| child_adoles_smoker | -0.11022 | 0.273707 | 0.795972 | 0.151579 | - | - | - | - | - | - |
| ENSG00000260070 | - | - | 2.204858 | - | - | 0.504971 | - | 0.722424 | - | 0.591821 |
| ENSG00000253005 | - | - | -1.92225 | - | - | -0.09475 | - | 0.166858 | - | -0.33667 |
| ENSG00000237757 | - | - | 0.231318 | - | - | -0.08343 | - | 0.485796 | - | 0.256828 |
| ENSG00000277692 | - | - | -1.28395 | - | - | -0.74946 | - | -0.54482 | - | -0.65752 |
| ENSG00000273437 | - | - | 0.876228 | - | - | 0.300186 | - | -0.0933 | - | 0.402451 |
| prs | - | - | - | 1.098038 | - | - | 0.443394 | - | 0.260079 | 0.415765 |
